# The therapeutic efficacy of adjunct therapeutic plasma exchange for septic shock with multiple organ failure: a single-center experience

**DOI:** 10.1186/s13054-020-03241-6

**Published:** 2020-08-24

**Authors:** Philip D. Keith, Adam H. Wells, Jeremy Hodges, Stephen H. Fast, Amber Adams, L. Keith Scott

**Affiliations:** 1 Critical Care Medicine, Lexington Medical Center, 2720 Sunset Boulevard, West Columbia, SC 29169 USA; 2 Critical Care Medicine, Novant Health Forsyth Medical Center, 3333 Silas Creek Parkway, Winston-Salem, NC 27103 USA; 3 Clinical Pharmacist, Novant Health Forsyth Medical Center, 3333 Silas Creek Parkway, Winston-Salem, NC 27103 USA; 4 Department of Mathematics, Limestone College, 1115 College Drive, Gaffney, SC 29340 USA; 5 Emergency Department Clinical Pharmacist, Cabell Huntington Hospital, 1340 Hal Greer Boulevard, Huntington, WV 25701 USA; 6 Division of Trauma and Surgical Critical Care, Louisiana State University Health Sciences Center, 1501 Kings Highway, Shreveport, LA 71103 USA

**Keywords:** Septic shock, Sepsis, Multiple organ failure, MODS, Plasma exchange, Plasmapheresis

## Abstract

**Background:**

Sepsis remains a common condition with high mortality when multiple organ failure develops. The evidence for therapeutic plasma exchange (TPE) in this setting is promising but inconclusive. Our study aims to evaluate the efficacy of adjunct TPE for septic shock with multiple organ failure compared to standard therapy alone.

**Methods:**

A retrospective, observational chart review was performed, evaluating outcomes of patients with catecholamine-resistant septic shock and multiple organ failure in intensive care units at a tertiary care hospital in Winston-Salem, NC, from August 2015 to March 2019. Adult patients with catecholamine-resistant septic shock (≥ 2 vasopressors) and evidence of multiple organ failure were included. Patients who received adjunct TPE were identified and compared to patients who received standard care alone. A propensity score using age, gender, chronic co-morbidities (HTN, DM, CKD, COPD), APACHE II score, SOFA score, lactate level, and number of vasopressors was used to match patients, resulting in 40 patients in each arm.

**Results:**

The mean baseline APACHE II and SOFA scores were 32.5 and 14.3 in TPE patients versus 32.7 and 13.8 in control patients, respectively. The 28-day mortality rate was 40% in the TPE group versus 65% in the standard care group (*p* = 0.043). Improvements in baseline SOFA scores at 48 h were greater in the TPE group compared to standard care alone (*p* = 0.001), and patients receiving adjunct TPE had a more favorable fluid balance at 48 h (*p* = 0.01). Patients receiving adjunct TPE had longer ICU and hospital lengths of stay (*p* = 0.003 and *p* = 0.006, respectively).

**Conclusions:**

Our retrospective, observational study in adult patients with septic shock and multiple organ failure demonstrated improved 28-day survival with adjunct TPE compared to standard care alone. Hemodynamics, organ dysfunction, and fluid balance all improved with adjunct TPE, while lengths of stay were increased in survivors. The study design does not allow for a generalized statement of support for TPE in all cases of sepsis with multiple organ failure but offers valuable information for a prospective, randomized clinical trial.

## Background

Sepsis remains a common condition accounting for nearly 1.3 million US hospitalizations, including 25% of ICU admissions annually [1]. Despite an overall improvement with early goal-directed therapy, septic shock remains the most common cause of death in non-coronary intensive care units with mortality rates approaching 70% when multiple organ failure develops [2].

Investigators have gained an understanding of sepsis as a complex interaction of cytokine storm, systemic inflammation, endothelial dysfunction, capillary leak, and pathologic hemostasis similar to thrombotic thrombocytopenic purpura (TTP) [3–7]. When fulminant, the end result is disseminated microcirculatory thrombosis resulting in tissue hypoxia, multiple organ failure, and death [8]. Steroids, activated protein C, plasma filtration, ascorbic acid, polymyxin B hemoperfusion, and thrombomodulin have all been investigated as a therapy for specific components of this pathway, but have largely failed to improve outcomes in clinical trials [9–15]. While each of these therapies may benefit individual patients with sepsis, the heterogeneity of sepsis syndrome makes it unlikely that any intervention targeting a single component of the pathway would be successful when utilized universally.

Therapeutic plasma exchange (TPE) may offer a unique treatment for sepsis, with its proposed action at multiple levels of this complex pathway. The initial cytokine storm leads to global inflammation and disruption of the endothelium leading to vasodilation, capillary leak, and activation of the coagulation cascade [3, 4, 7, 16, 17]. While plasma filtration has been demonstrated to lower circulating levels of many of these mediators in both experimental and clinical studies [17–21], trials investigating survival with various forms of hemofiltration and cytokine binding have yielded inconsistent results [18, 19, 21, 22].

Successful treatment of sepsis appears to require more than rebalancing inflammatory mediators, and TPE may offer further benefit by offsetting the effects of endothelial dysfunction. Far from a passive conduit, the endothelium plays a major role in the sepsis pathway and has become a common target for therapy. Hypotension results not only from inflammatory vasodilation, but also from increased vascular permeability resulting from endothelial injury [3–6, 17]. Studies in septic and hemorrhagic shock have identified circulating markers of endothelial injury, which have been associated with electron microscopic changes to the endothelium, and increased mortality [5, 17, 23]. Resuscitation with fresh frozen plasma (FFP) has shown restoration of endothelial integrity, as assessed by improved levels of these circulating markers and improved microscopic appearance of the endothelium [17, 23]. In cases of massive hemorrhage, mortality has improved with a transfusion strategy including FFP [23]. These findings may partially explain the clinical improvement that is often seen with TPE when using fresh frozen plasma as the replacement fluid.

Another clinical effect of endothelial activation is pathologic activation of the clotting cascade leading to a hypercoagulable microcirculatory state. Decreased ADAMTS-13 activity and increased ADAMTS-13 inhibitors are prevalent leading to increased thrombogenic ultra-large von Willebrand factor (ULvWF) multimers, resulting in diffuse microcirculatory platelet thrombosis. Increased plasminogen activator inhibitor (PAI-1) activity leads to decreased fibrinolysis and disseminated fibrin-rich microcirculatory clotting. The net result is a non-consumptive, platelet- and fibrin-rich microcirculatory thrombotic state with non-specific coagulation findings, often distinct from DIC, TTP, and HUS [3, 7, 16, 24, 25]. Activated protein C and thrombomodulin are among the therapies that have been investigated to reverse this process, without success [9, 10, 15]. Plasma exchange is unique in that it addresses both the pathologic coagulation cascade and platelet dysfunction by removing the ULvWF multimers, ADAMTS-13 inhibitors, and PAI-1 while restoring ADAMTS-13 activity [7].

Case reports, case series, meta-analyses, and a single adult prospective, randomized clinical trial have yielded inconsistent results on the efficacy of TPE for sepsis [16, 18, 22, 26, 27]. Based on the available data, the American Society for Apheresis (ASFA) offers a category III, 2B recommendation for the use of TPE for sepsis with multiple organ failure, allowing for individualized use on a case to case basis [2, 28].

Using this recommendation as a guide, our institution has utilized TPE in select cases of catecholamine refractory septic shock with multiple organ failure. By incorporating markers of poor outcome [29–36] as guidelines for consideration for TPE, we have sought to identify those patients with the clinical phenotype unlikely to survive with standard therapy alone (Table 1). In our retrospective, observational study, we analyzed data from the electronic medical record and compared outcomes in patients meeting these criteria who received adjunct TPE to propensity-matched patients meeting the same criteria who received standard therapy alone.

**Table 1:** Study population

**Inclusion criteria: 1, 2, and 3 plus A, B, C, or D**	
1. New known or suspected infection (with a chance for source control if applicable)	A. Lactic acidosis and/or failure of lactic acid clearance
2. Multiorgan failure (≥ 2 organs failing)	B. Worsening acidosis despite adequate fluid resuscitation and/or dialysis
3. Two or more pressors, rapidly rising pressor needs, and/or inability to wean pressors^a^	C. Mottling skin appearance despite appropriate resuscitation
	D. Acute drop in platelet count (± thrombocytopenia)
**Exclusion criteria**	
Cardiogenic shock	Active metastatic malignancy
Hemorrhagic shock	Limitations to aggressive care
Ischemic colitis without surgery	Planned withdrawal of care
Cardiac arrest at presentation	

^a^Hypotension must be due to sepsis

## Methods

### Study design

This retrospective, observational study on the effect of TPE as an adjunct therapy for septic shock with multiple organ failure was conducted by reviewing the electronic medical records of adult patients, 18 years old and older, treated for septic shock at Novant Health Forsyth Medical Center from August 2015 to March 2019. The study was approved by the Institutional Review Board at Novant Health Forsyth Medical Center. Informed consent was not required as the study reports observational, retrospective data obtained from chart review.

### Study subjects

The intervention group patients were identified via the electronic medical record to include patients with the primary diagnosis of septic shock and a procedure code for apheresis during the specified time frame. Forty patients were identified who received at least one TPE treatment and met the criteria in Table 1.

The control group patients were identified using report filters meeting our institutional guidelines for the consideration of TPE in patients with sepsis within the same time frame as the intervention group. Patients with the primary diagnosis of shock plus each of the following flags were screened: 2 or more vasopressors, lactic acid > 2 mmol/L, platelet nadir < 200 × 10^3^/μL, and pH < 7.3. A total of 160 patients were identified. Two study researchers independently screened each patient for the criteria in Table 1, excluding 117 patients. Data was collected on the remaining 43 patients, and propensity matching was performed as discussed below.

### Intervention

All patients in both groups were treated for sepsis at the discretion of the attending intensivist. All patients were ordered to receive 30 cc/kg of IV fluids and timely administration of empiric antibiotics while in the emergency department, prior to admission to the hospital, per the hospital’s sepsis protocol. While this sepsis treatment protocol was available, individualized treatment occurred in both groups based on physician preferences (e.g., adjunct steroids, ascorbic acid, thiamine). All mechanically ventilated patients were managed with a lung-protective strategy according to the ARDSnet protocol. In cases of severe respiratory acidosis, adjustments to the ventilator were made according to ARDSnet recommendations, allowing for permissive hypercapnia when appropriate. In cases of severe, life-threatening acidosis, ventilator settings may have been adjusted outside this protocol by the attending physician.

In our institution, plasma exchange is performed by the nephrology department, and the decision to perform TPE involved a multidisciplinary approach. The attending intensivist may have requested TPE if patients met the criteria outlined in Table 1. The nephrology service was then consulted and reviewed each case. If the consulting nephrologist agreed that TPE would potentially benefit the patient, then TPE would be performed. If the nephrologist did not agree that TPE was indicated, then the patient did not receive TPE as part of their care.

In patients undergoing TPE, vascular access was obtained by venous insertion of a 14-French double-lumen temporary hemodialysis catheter. TPE was performed using 120% of the calculated total plasma volume, adjusting for obesity. Fresh frozen plasma was used as a replacement fluid in all cases. In patients requiring continuous renal replacement therapy, dialysis was interrupted for the duration of TPE. The number of treatments was not standardized; rather, daily treatments were performed until the treating team (a) felt that the patient reached maximum benefit, (b) after a set number of treatments per physician preference (similar to TTP protocol), or (c) the patient clinically deteriorated and the treating physician felt that further treatment was not clinically warranted, treatment was felt to be medically futile, and/or surrogate decision-makers wished to transition to comfort measures. A majority of providers followed the treatment protocol of Busund et al. [16], performing a single treatment followed by a second treatment the following day if the clinical condition did not improve.

### Definition of variables

The primary study outcome was all-cause 28-day mortality. Secondary outcomes included hospital mortality, a new need for renal replacement therapy (RRT) during admission and at discharge, mortality associated with a new need for renal replacement therapy, ICU length of stay, hospital length of stay, daily fluid balance, and change in SOFA and cardiac SOFA scores 48 h after identification in patients surviving at least 48 h. “Time zero” for the intervention group was defined as the documented date and time of completion of the first plasma exchange treatment. “Time zero” for controls was defined as the first recorded vital signs in the intensive care unit. Patients were propensity-matched using age, gender, chronic co-morbidities (HTN, DM, CKD, COPD), APACHE II score, SOFA score, lactate level, and number of vasopressors at ICU admission, while all primary and secondary outcomes were measured and calculated based on time zero defined above.

Patient charts were reviewed through hospital discharge or death. For patients discharged prior to day 28, mortality was assessed by searching subsequent admissions and online obituaries. Values used for calculation of the 48-h SOFA scores were the most recent vital signs and labs to the exact hour of inclusion. Patients who expired prior to 48 h were excluded from the SOFA and fluid balance analyses.

### Computation and matching of propensity score

Patients in the intervention and control groups were propensity-matched using age, gender, chronic co-morbidities (HTN, DM, CKD, COPD), APACHE II score, SOFA score, lactate level, and number of vasopressors at ICU admission to generate propensity scores.

### Patient characteristics

The study included 80 patients with 40 in each arm. Baseline patient demographics are summarized in Table 2. Patients in both arms had a high mortality risk with similar baseline APACHE II and SOFA scores. While baseline SOFA scores were similar, patients in the intervention arm had higher SOFA scores at time zero. All patients presented with septic shock requiring at least two vasopressors, and a majority required a new start of renal replacement therapy. Patients in the two arms differed by ventilator requirement at inclusion with patients in the intervention group requiring ventilator support more frequently than those in the control group (*p* = 0.014). We noted no other differences in baseline characteristics though the mean age was numerically higher in the control group (*p* = 0.077).

**Table 2:** Baseline characteristics of 80 matched patients included in the trial

Variable	TPE (***n*** = 40)	Standard care (***n*** = 40)	***p***
Gender (M/F)	24/16	21/19	0.65
Mean age (years)	57.6 ± 13.4	63.6 ± 16.3	0.077
Septic shock^a^	40 (100%)	40 (100%)	1
Ventilator requirement	39 (97.5%)	29 (72.5%)	0.003
ESRD	3 (7.5%)	3 (7.5%)	1
Mean APACHE II	32.5 ± 6.0	32.7 ± 7.2	0.88
Mean SOFA on admission	14.3 ± 3.6	13.8 ± 2.4	0.426
Mean SOFA at time zero^b^	15.8 ± 2.9	13.8 ± 2.4	0.001
Hypertension	21	26	0.364
Chronic kidney disease	10	10	1
Diabetes mellitus	15	17	0.82
COPD	8	8	1
Lactic acid at time zero^b^	8.1 ± 6.6	6.6 ± 4.7	0.219
Number of pressors at time zero^b^	3.1 ± 0.76	2.9 ± 0.83	0.263
**Site of infection**			0.328
Pneumonia	23	17	
GU	6	8	
GI/biliary	6	6	
Skin/soft tissue	1	4	
Endocarditis	3	1	
Primary bacteremia	1	4	

^a^All patients included were on at least two vasopressors per selection criteria

^b^TPE “time zero” is the time of the first TPE; standard care “time zero” is the hour of the first recorded vital signs in ICU

### Statistical analyses

Statistical analysis was performed by an independent statistician, using XLSTAT by Addinsoft (Windows version) and Xrealstats from http://www.real-statistics.com/ (Windows version) add-ins for Microsoft Excel. Univariate comparisons of baseline characteristics were made by unpaired *t* test for continuous variables and Fisher’s exact test for categorical variables. The chi-square test was used to test the differences in infectious origin between the two groups. Changes in APACHE 2 and SOFA from baseline within a group were assessed by paired *t* test. Fisher’s exact test was used to test the differences in survival between the groups. For survival analysis, a Kaplan-Meier estimate is provided using the log-rank test to compare cumulative survival. Data is presented as mean ± standard deviation.

## Results

**Fig. 1:**
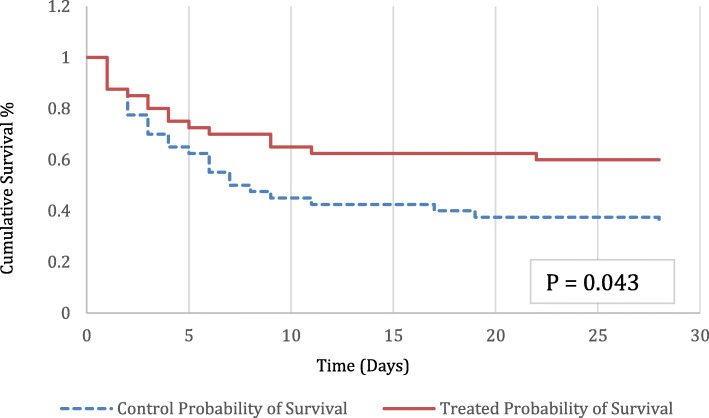
Twenty-eight-day survival in patients with septic shock and multiple organ failure receiving TPE in addition to standard therapy (*n* = 40) or standard therapy alone (*n* = 40) (*p* = 0.043)

Clinical outcomes are summarized in Fig. 1 and Table 3. The overall 28-day mortality rate was 40% in the intervention group versus 65% in the control group (*p* = 0.043). The relative risk reduction for mortality was 38.5%. In this study population, one additional life would be saved for every four patients treated with TPE. Hospital mortality was 42.5% with TPE compared to 65% with standard care alone (*p* = 0.072). Table 4 reports mortality by primary site of infection and isolated pathogens. The subgroup of patients with pneumonia as the primary site who received adjunct TPE demonstrated the greatest improvement in 28-day mortality compared to patients with pneumonia who received standard care alone (47.8% vs. 88%, *p* = 0.017). Collectively, other sites of infection demonstrated 29.4% mortality with adjunct TPE compared to 48% mortality with standard therapy alone (*p* = 0.332). Additional subgroup analyses were not possible due to the small sample size in each of the other sites of infection. Changes in SOFA scores at 48 h showed improvement from baseline in the TPE group compared to standard care alone, in those patients surviving at least 48 h (*p* <  0.001). Patients receiving adjunct TPE had a more favorable fluid balance at 48 h, as well (*p* = 0.01), Table 3. Patients undergoing adjunct TPE required initiation of renal replacement therapy in 67.6% of cases, compared to 51.4% in those receiving standard care alone (*p* = 0.236). The mortality associated with new RRT was 48% in those receiving TPE compared to 79% in those receiving standard of care alone (*p* = 0.06), while there was no difference in the new need for renal replacement therapy at discharge in survivors. Both ICU and hospital lengths of stay were longer in patients receiving TPE (*p* = 0.003, *p* = 0.006).

**Table 3:** Primary and secondary outcomes

Outcome	TPE (*n* = 40)	Standard care (*n* = 40)	*p*
**28-day mortality**			
Total study population	16 (40%)	26 (65%)	0.043
Pneumonia group	11/23 (47.8%)	15/17 (88%)	0.017
Other groups	5/17 (29.4%)	11/23 (48%)	0.332
Hospital mortality	17 (42.5%)	26 (65%)	0.072
Baseline SOFA in 48 h survivors^a^	15.7 ± 3.0	13.2 ± 2.2	< 0.001
SOFA at 48 h^a^	12.6 ± 4.5	12.9 ± 3.7	0.782
Change in SOFA^a^	3.1 ± 2.6	0.32 ± 3.5	< 0.001
Baseline Cardiac SOFA in 48 h survivors^a^	4.0 ± 0.17	3.8 ± 0.73	0.336
Cardiac SOFA at 48 h^a^	1.5 ± 1.54	2.8 ± 1.6	0.001
Change in cards SOFA^a^	2.47 ± 1.52	1.03 ± 1.52	< 0.001
New need for RRT during admission^b^	25 (67.6%)	19 (51.4%)	0.236
Mortality associated with new RRT	12 (48%)	15 (79%)	0.06
New need for RRT at d/c in survivors	4 (30.8%)	1 (25%)	1
Net daily fluid balance preceding 24 h	4304 ± 2900	5269 ± 3629	0.244
Net daily fluid balance after 48 h	− 78 ± 1837	1466 ± 2675	0.01
Change in fluid balance	− 4382 ± 2958	− 3803 ± 4431	0.542
ICU LOS^c^	16.6 ± 15.8	8.0 ± 7.4	0.003
Hospital LOS^c^	24.6 ± 22.4	13.0 ± 13.2	0.006

^a^For patients who survived at least 48 h (*n* = 34 for the TPE arm and *n* = 31 for the standard arm)

^b^Three ESRD in each group

^c^Note that the standard care arm experienced more 28-day mortality

**Table 4:** Mortality associated with infection site and pathogen

	Overall	TPE	No TPE	*p*
**Site of infection**				
Pneumonia	26/40 (65%)	11/23 (47.8%)	15/17 (88.2%)	0.017
GU	2/14 (14.3%)	0/6 (0%)	2/8 (25%)	
GI/biliary	6/12 (50%)	2/6 (33.3%)	4/6 (66.7%)	
Skin/soft tissue	1/5 (20%)	1/1 (100%)	0/4 (0%)	
Endocarditis	2/4 (50%)	1/3 (33.3%)	1/1 (100%)	
Primary bacteremia	5/5 (100%)	1/1 (100%)	4/4 (100%)	
**Organism cultured**				
MRSA	2/5 (40%)	1/3 (33.3%)	1/2 (50%)	
*Streptococcus*	3/5 (60%)	1/2 (50%)	2/3 (66.7%)	
*E. coli*	6/11 (54.5%)	2/5 (40%)	4/6 (66.7%)	
*Pseudomonas*	2/2 (100%)	1/1 (100%)	1/1 (100%)	
*Enterococcus*	1/2 (50%)	0/1 (0%)	1/1 (100%)	
*Enterobacter*	3/8 (37.5%)	2/3 (66.7%)	1/5 (20%)	
*Klebsiella*	2/6 (33.3%)	0/4 (0%)	2/2 (100%)	
Influenza	2/3 (66.7%)	0/1 (0%)	2/2 (100%)	
*Serratia*	1/1 (100%)	1/1 (100%)	n/a	
*C. difficile*	1/2 (50%)	n/a	1/2 (50%)	
*Salmonella*	0/1 (0%)	n/a	0/1 (0%)	
Culture negative	3/14 (21.4%)	1/10 (10%)	2/4 (50%)	
Polymicrobial	16/20 (80%)	7/9 (77.8%)	9/11 (81.8%)	

*Pathogens in polymicrobial infections are not specified in the numbers above

Labs were ordered at the discretion of the attending physician, as part of the standard therapy, and retrospectively analyzed (Tables 5, 6, and 7). Initial lactate levels were similar in both groups, 8.1 vs. 6.6 (*p* = 0.219), but were lower at 24 h in those receiving adjunct TPE (4.8 vs. 6.9 *p* = 0.145), Table 5. Lower levels at 24 h were associated with decreased mortality in both groups (2.9 in TPE survivors vs. 7.2 in TPE deaths (*p* = 0.048); 4.4 in control survivors vs. 8.2 in control deaths (*p* = 0.05)) (Table 6). Platelet count at enrollment was lower in those receiving adjunct TPE (102.6 vs. 172.8, *p* <  0.001), and platelet nadir was also lower in this group (49.7 vs. 73.7, *p* = 0.008) (Table 7). Within both groups, lower nadir levels trended towards increased mortality (Table 6). The resolution of thrombocytopenia was associated with improved mortality in both groups, while failure to recover was associated with increased mortality in both groups (Table 7).

**Table 5:** Effect of TPE on objective measures of organ dysfunction compared to controls

Measure	TPE (*n* = 40)	Standard care (*n* = 40)	*p*
Cards SOFA at time zero^a^	4.0 ± 0.16	3.9 ± 0.65	0.348
Cards SOFA at 48 h	1.5 ± 1.54	2.8 ± 1.6	0.001
Lactate at time zero^a^	8.1 ± 6.6	6.6 ± 4.7	0.219
Lactate at 24 h	4.8 ± 5.9 (*n* = 29)	6.9 ± 5.5 (*n* = 35)	0.145
Platelet count at time zero^a^	102.6 ± 68.5	172.8 ± 72.7 (*n* = 37)	< 0.001
Platelet nadir	49.7 ± 36.6	73.7 ± 41.9	0.008
P/F ratio at time zero^a^	176.3 ± 139.2 (*n* = 38)	161.8 ± 113.3 (*n* = 33)	0.631
P/F ratio at 48 h	217 ± 100 (*n* = 29)	223.6 ± 144.3 (*n* = 30)	0.838
Extubations	3	1	
New intubations	0 (1 placed on ECMO)	5	
Deaths prior to 48 h	6	9	0.568

^a^TPE “time zero” is the time of the first TPE; standard care “time zero” is the hour of the first recorded vital signs in ICU

Lactic acid measured mmol/L; platelet count measured × 1000/μL

**Table 6:** Effect of TPE on lactate and platelets with associated mortality

Measure	Survivors	Non-survivors	*p*
Platelet nadir, TPE (× 1000/μL)	58.1	37.2	0.076
Platelet nadir, control (× 1000/μL)	83.8	68.2	0.27
Platelet recovery, TPE^a^	23/25 (92%)	2/25 (8%)	0.0001
Lack of platelet recovery, TPE^a^	1/15 (6.7%)	14/15 (93.3%)	
Platelet recovery, control^a^	13/19 (68.4%)	6/19 (31.6%)	0.0003
Lack of platelet recovery, control^a^	1/16 (6.2%)	15/16 (93.8%)	
24 h lactate levels, TPE	2.9	7.2	0.048
24 h lactate levels, control	4.4	8.2	0.05

^a^Recovery to > 100 × 10^3^/μL; note: five control patients died prior to developing platelet count < 100 × 10^3^/μL

**Table 7:** Platelet/coagulation profile

	Enrollment platelet count (× 1000/μL)	Nadir platelet count (× 1000/ μL)	Nadir days	Platelet recovery (> 100 × 10^3^/μL) (*n*)	Baseline ADAMTS-13 activity* (%)
TPE (*n* = 40)	102.6	49.7	4.7	25	42
Controls (*n* = 40)	172.8	73.7	3.4	19	42
Survivors (*n* = 38)	169.9	67.5	3.7	36	43.7
Non-survivors (*n* = 42)	167.2	58.2	2.5	8	40.1

*ADAMTS-13 levels were not routinely collected unless concern for TTP (*n* = 43)

ADAMTS13 levels were not routinely assessed but were similar in patients receiving TPE and standard care, 42% in both groups. Similarly, there was no difference in levels of survivors compared to non-survivors (43.7% vs. 40.1%), Table 7. Serial levels were not checked in either group.

Thirty-nine patients receiving TPE required mechanical ventilation at enrollment, with an average PaO_2_/FiO_2_ (P/F) ratio of 176.3, while 29 patients receiving standard therapy alone required mechanical ventilation at enrollment, with an average P/F ratio of 161.8. In the initial 48 h, 3 patients receiving TPE were extubated, compared to one patient receiving standard care. There was no difference in the P/F ratio between the groups at 48 h (217 vs. 223.6, *p* = 0.838). Five new patients required intubation in the standard care arm within the first 48 h, while one patient receiving TPE was placed on ECMO.

There were no documented adverse events attributed to temporary hemodialysis catheter placement or the TPE procedure.

## Discussion

The results of our study suggest a benefit and potential role for adjunct TPE in the treatment of sepsis with multiple organ failure. Prospective trials on this topic are lacking, and our results are among the largest in a body of evidence largely built on individual case reports and series. The 25% absolute reduction in mortality meets statistical significance and strongly suggests clinical benefit. The overall mortality is high in our study, but consistent with historical rates when adjusted for severity of illness (73–95.2% based on admission APACHE II and SOFA scores). Patients in both arms of our study had multiple comorbid conditions that increased mortality risk independent of sepsis, including hypotension requiring multiple vasopressors, acute renal failure, and moderate ARDS. The results and the effect of TPE on outcomes are very similar to those seen in the prospective trial performed by Busund et al. in a similar patient population [16].

In addition, patients receiving TPE in our study had improved SOFA and cardiac SOFA scores at 48 h, indicating improved organ function and hemodynamics. While the predicted mortality based on SOFA scores may be overstated, trends in SOFA scores serve as valuable predictors of outcomes [37–39]. Fortenberry and colleagues reported improvement in organ dysfunction (as reflected by changes in PELOD scores from baseline) and 28-day mortality in septic pediatric patients meeting similar criteria who received TPE [37]. The favorable fluid balance seen in patients receiving TPE was also noteworthy. This finding may be explained by endothelial stabilization, leading to improved hemodynamics and less need for volume resuscitation. In a retrospective study, where no research labs were collected, this cannot be proven, and future prospective studies should consider the evaluation of endothelial markers. Nevertheless, the improved hemodynamic profile, organ function, and favorable fluid balance are all associated with improved outcomes and encourage further studies [40–43].

The coagulopathy of sepsis is quite complex, composed of platelet dysfunction and abnormalities of the coagulation cascade. Thrombocytopenia, DIC, and decreased ADAMTS-13 activity have all been associated with poor outcomes in sepsis [25, 31, 33, 34, 44]. We believe that coagulopathy is often present clinically, prior to laboratory derangements, so absolute values were not used to determine candidacy for treatment at our institution or to monitor response to treatment. Retrospective analysis of platelet count did demonstrate more favorable outcomes in patients with higher platelet counts and resolution of thrombocytopenia, while lower platelet counts and failure of platelet recovery were associated more commonly with death (Table 6). We did not routinely measure markers of the coagulation cascade except in patients on anticoagulation, so we were unable to assess these values in our study. Interestingly, baseline ADAMTS-13 levels were similar in all patients in both arms, suggesting a possible association with severity of illness, as suggested in prior reports with sepsis [8, 25]. We did not measure serial levels, so it is unclear whether TPE helped to restore activity, and if so, if this restoration was associated with improved outcomes. While none of these findings affected the clinical treatment of patients in this study, the data may serve useful in future prospective trials.

Our study has limitations beyond those common to small, retrospective, single-center studies. First, the difference in time zero in the two arms potentially introduces bias. In a retrospective study, the intervention is easily defined, but since the control group did not receive treatment, we had to define an arbitrary time zero. Time zero for the intervention group was defined as the time of documentation of the initial TPE completion (regardless of ICU admission date and time). For control patients, time zero was defined as the time of the first recorded ICU vital signs. To limit bias, patients were propensity matched based on age, gender, number of vasopressors, lactate levels, chronic comorbidities, and APACHE II and SOFA scores on ICU admission. SOFA scores were also calculated at time zero and were higher in the intervention group compared to the control group, predicting a higher mortality in this group (Table 2), (*p* = 0.001).

While our institution does have a sepsis protocol, individual variation exists among providers. This variability is unlikely to influence outcomes, as multiple trials have demonstrated no difference in mortality using various resuscitation strategies [45, 46]. In addition, since both the control group and intervention group were cared for by the same providers during the same time frame, variation between the groups should be similar.

The decision to utilize TPE was provider dependent and involved an interdisciplinary approach between the attending intensivist and nephrologist. General guidelines were developed (Table 1), but screening did not occur, and TPE was not considered unless the attending intensivist felt that it might be beneficial. Therefore, some patients that may have been candidates for TPE were likely not considered for treatment and likely fell into our control group. Furthermore, meeting the criteria did not guarantee that TPE would be provided. Ultimately, the decision was made by the consulting nephrologist on a case-to-case basis. A large majority of TPE for sepsis was prescribed by a small number of providers within both groups. This bias cannot be eliminated from a retrospective study where providing the intervention is not randomized, but using clearly defined, objective inclusion and exclusion requirements allows for matching and statistical comparison.

Another limitation of our study was the lack of uniformity in the duration of treatment in the intervention group. While most patients received between one and five treatments (92.5%), no objective guidelines were established at our facility to standardize the duration of TPE. Of the three patients receiving more than 5 treatments, two had prolonged admissions and received two separate courses of TPE, with different inciting infections. The third received treatment until normalization of platelets, based on provider preference. Efficacy and duration were most often guided by the hemodynamic response and lactate clearance. Many providers stopped TPE after vasopressor needs resolved, while others preferred a standing order for 3 or 5 treatments. Lactic acid levels declined more rapidly in patients receiving TPE, and levels were lower at 24 h in survivors in both groups (Tables 5 and 6). Whether additional treatments would further enhance lactate clearance and improve mortality cannot be determined but should be a priority in future, prospective trials.

ICU and hospital lengths of stay were longer in the intervention group but may not be reflective of true morbidity or cost as the standard care group had more early deaths. Additionally, more patients receiving TPE required new start renal replacement therapy, but the mortality associated with this treatment was clinically less in the TPE group (48% vs. 78%, *p* = 0.06). There was no difference in the new need for RRT at discharge in survivors in our study. A larger sample size and longer follow-up interval are needed to assess the true impact on morbidity, resources, and long-term system costs.

The retrospective design of the study was not optimal for detecting adverse events associated with TPE. All patients in our study were, by definition, hemodynamically unstable. It is impossible to attribute hemodynamic instability to TPE or to exclude TPE as a contributing factor based on our review of documentation. There were no recorded complications attributed to temporary dialysis catheter placement, and no TPE treatments were aborted for clinical deterioration. However, we cannot exclude other potential adverse events that were unable to be tracked or identified. The potential adverse effects of TPE are well documented [47], and a recent pilot study demonstrated the feasibility and safety of early TPE in a similar patient population, reporting no adverse events [17]. Nevertheless, a prospective, randomized trial should serve to identify potential adverse events associated with TPE specifically in the adult sepsis population.

The results of our study are encouraging but limited by design, and the results cannot be used to change existing standards for the treatment of sepsis. The information gained from our experience offers valuable information and should be used to assist with the design of a multicenter, randomized, controlled trial to better assess this potentially useful intervention.

## Conclusions

TPE has been proposed as a therapeutic option for sepsis, but inadequate trial data exists to support or refute its efficacy in this patient population. Our results add to the body of evidence that support TPE in a subset of adult patients with sepsis. A multicenter, prospective, randomized controlled trial is needed to investigate the efficacy of TPE in adult patients with septic shock with multiple organ failure.

## Data Availability

The datasets used and/or analyzed during the current study are available from the corresponding author on reasonable request.

## Abbreviations

ADAMTS-13: von Willebrand factor-cleaving protease
APACHE: Acute Physiology and Chronic Health Evaluation
ARDS: Acute respiratory distress syndrome
ASFA: American Society for Apheresis
DIC: Disseminated intravascular coagulation
FFP: Fresh frozen plasma
HUS: Hemolytic uremic syndrome
ICU: Intensive care unit
PA-1: Plasminogen activator inhibitor-1
RCT: Randomized controlled trial
RRT: Renal replacement therapy
SOFA: Sequential Organ Failure Assessment
TPE: Therapeutic plasma exchange
TTP: Thrombotic thrombocytopenic purpura
ULvWf: Ultra-large von Willebrand factor
